# A novel mutation in GAS8 gene associated with chronic rhinosinusitis with nasal polyposis in a case of primary ciliary dyskinesia: a case report

**DOI:** 10.3389/fped.2024.1345265

**Published:** 2024-05-30

**Authors:** Maria Cristina Artesani, Sara Santarsiero, Emanuela Sitzia, Francesca Romana Lepri, Monia Magliozzi, Fabio Majo, Nicola Ullmann, Alessandra Stracuzzi, Antonio Novelli, Giovanni Cristalli, Alessandro Fiocchi

**Affiliations:** ^1^ Allergy Unit, Bambino Gesù Children’s Hospital, IRCCS, Rome, Italy; ^2^ Otorhinolaryngology Unit, Bambino Gesù Children’s Hospital, IRCCS, Rome, Italy; ^3^ Laboratory of Medical Genetics, Translational Cytogenomics Research Unity, Bambino Gesù Children Hospital, IRCCS, Rome, Italy; ^4^ Paediatric Pulmonology and Cystic Fibrosis Unit, Bambino Gesù Children’s Hospital, IRCCS, Rome, Italy; ^5^ Pathology Unit, Bambino Gesù Children’s Hospital, IRCCS, Rome, Italy

**Keywords:** chronic rhinosinusitis with nasal polyposis (CRSwNP), primary ciliary dyskinesia (PCD), GAS8, otitis, bronchiectasis

## Abstract

**Background:**

Primary ciliary dyskinesia (PCD) is considered a rare cause of chronic rhinosinusitis with nasal polyposis (CRSwNP), which is reported in 6% of children with PCD. The forms of PCD associated with the variants of the GAS8 gene identified so far seem to be linked to recurrent respiratory infections (sinusitis, otitis, and bronchiectasis) without situs inversus.

**Case presentation:**

We report a case of an 11-year-old girl with recurrent otitis media, productive cough, and chronic rhinosinusitis with nasal polyposis with homozygosity for a novel nonsense mutation in the GAS8.

**Conclusion:**

Children with CRSwNP should be treated in a multidisciplinary manner (ENT, pulmonologist, allergist, pathologist, pediatrician, and geneticist) because nasal polyposis often hides etiologies that must be recognized.

## Introduction

The availability of new therapeutic options with biological treatments has attracted the attention of pediatric allergists to chronic rhinosinusitis with nasal polyposis (CRSwNP) (1). This disease, which with its recurrence leads school-age and adolescent children to the need for numerous surgical interventions, is associated with a series of clinical conditions, such as bronchial asthma, allergic rhinitis, allergic fungal rhinosinusitis, aspirin intolerance (Samter’s/Widal's triad), eosinophilic granulomatosis with polyangiitis (EGPA), non-allergic rhinitis with eosinophilia (NARES), cystic fibrosis (CF), and primary ciliary dyskinesia (PCD). PCD is considered a rare cause of CRSwNP, but CRSwNP is reported in 6% of children with PCD (mean age 10.4 years, range, 0.8–18 years) (2). This genetic disease is present in approximately 1 in 7,500 individuals worldwide (3) and is characterized by motile cilia dysfunction. The main symptoms are recurrent upper and lower respiratory tract infections with bronchiectasis and about half of patients with PCD have situs inversus due to dysfunction of motile embryonic nodal cilia. The ciliary ultrastructural phenotypes are highly heterogeneous because of the high complexity of ciliary axoneme (4). The ultrastructural configuration of human motile cilia is a “9 + 2” arrangement, consisting of nine outer doublets of microtubules and two central single microtubules, inner dynein arms (IDAs) and outer dynein arms (ODAs), radial spokes (RSs), and nexin-dynein regulatory complexes (N-DRCs). More than 50 genes encoding proteins involved in ciliogenesis, ciliary structure, and function are identified as PCD-causing (5). Among these, the most commonly involved in Europe and North America are DNAH5, DNAH11, DNAI1, CCD39, and CCD40 (3), which are associated with ODA defects (DNAH5, DNAH11, DNAI1) or with N-DRC defects (CCD39 and CCD40). N-DRC defects have also been associated with mutations in the GAS8 gene, which is located on chromosome 16q and contains 11 exons. GAS8 encodes DRC4, a component of the N-DRC, which plays an important structural and functional role in ciliary movement, although with very subtle abnormalities of ciliary beat and ultrastructure detectable by high-speed videomicroscopy and transmission electron microscopy (TEM) (4, 6).

Herein, we report a pediatric case of PCD caused by a novel homozygous mutation in the GAS8 gene.

## Case description

After obtaining informed consent from the patient’s parents, we report the case of an 11-year-old girl with consanguineous parents of Arab ethnicity referred to our Chronic Rhinosinusitis with Nasal Polyposis Multidisciplinary team. Clinical history identified upper respiratory airway recurrent infections from the first days of life, including persistent rhinitis with mucous and mucopurulent rhinorrhea and recurrent otitis media. At the age of 10 years, after an ENT evaluation with nasal endoscopy execution, bilateral CRSwNP was diagnosed, in the absence of apparent comorbidities, for which she had undergone functional endoscopic sinus surgery (FESS). Despite this, her symptoms persisted, including nasal obstruction, runny nose, and productive cough of recent onset.

Skin prick test (wheal ø 7 mm) and specific IgE (*Dermatophagoides farinae*, 1.67; *Dermatophagoides pteronyssinus*, 1.45 kUI/L) identified an allergy to dust mites. Environmental measures, oral antihistamines, and nasal steroids got only mild improvement in nasal symptoms.

Basal spirometry revealed an obstructive pattern with forced vital capacity (FVC) 75%, forced expiratory volume (FEV1) 58%, maximal mild-expiratory flow (MMEF) 85%, and maximal expiratory flow (MEF50) 57% of the expected normal value; the bronchodilation test did not show a significant change in lung volumes. Work-up after routine examination included a chest computed tomography scan, showing two parenchymal thickenings with a simultaneous aerial bronchogram consisting of cylindrical bronchiectasis, corresponding to the lateral segment of the middle lobe and the upper lingular segment.

According to our CRSwNP protocol, we evaluated the PICADAR score (7), which was 3, and nasal nitric oxide (nNO) (NIOX VERO® nasal), significantly reduced at 18 nl/mi (the mean value between the two nostrils).

The sweat chloride test was negative (Cl 24 mmol/L), as were genetic evaluations for CF.

**Figure 1:**
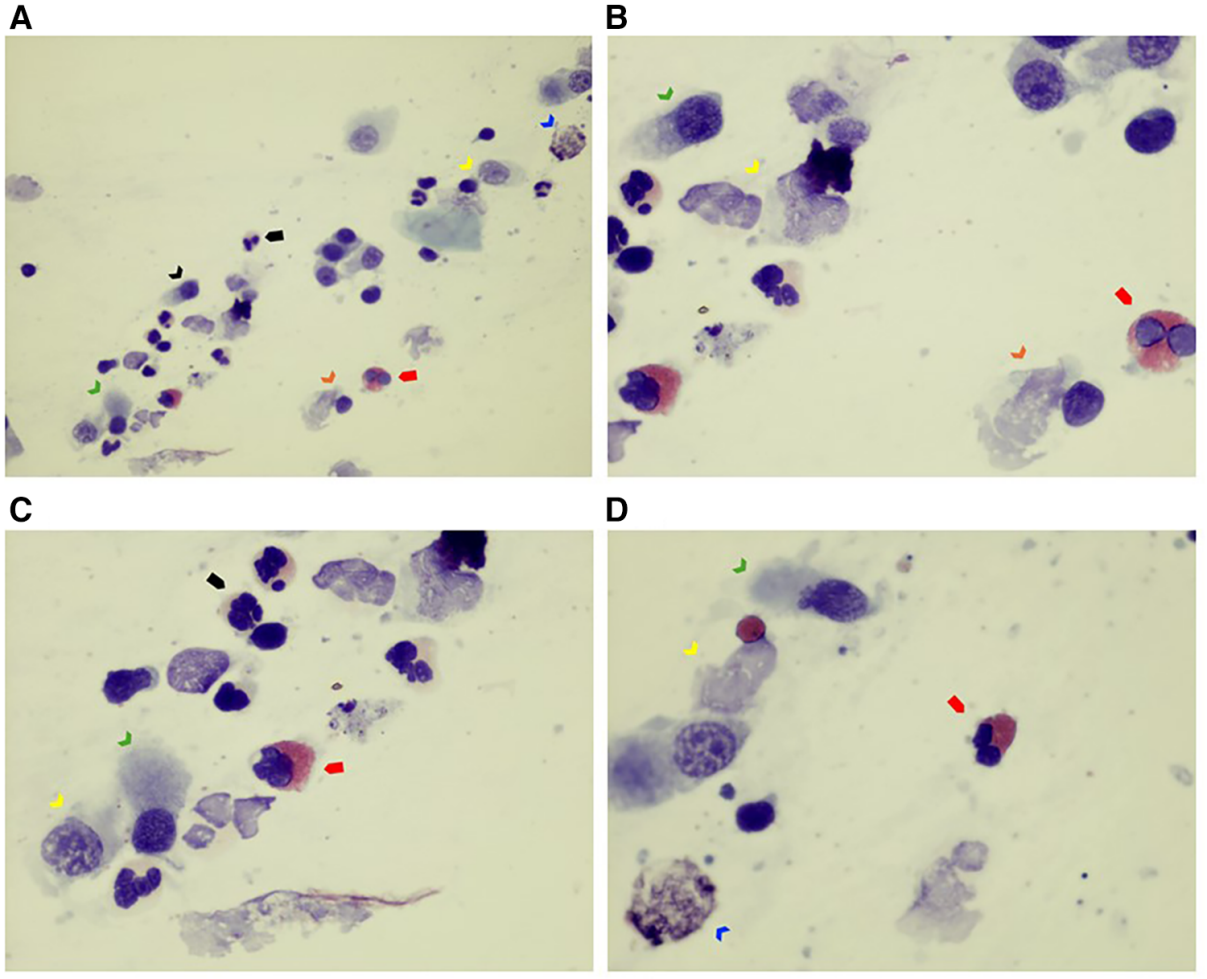
(**A**–**D**) Nasal cytology sample collected by nasal scraping. The sample of nasal epithelial cells is poor (ciliated cells) (see black arrow). Rare mucous cells (goblet cells) are visible (see blue arrow). Note that ciliated cells are almost all abnormal: the typical hyperchromatic supranuclear streak (HSS), normally present in healthy cells, as well as the ciliary apparatus, are poorly recognizable (see green arrow). Furthermore, degenerative and inflammatory features are noticeable (dyschromia and karyorrhexis of nucleus, and cytoplasmic vacuolization, see yellow arrow; ciliocytophthoria, see orange arrow). Some inflammatory cells such as eosinophils (see red full arrow) and neutrophils (see black full arrow) are present. May–Grünwald Giemsa (MGG) stain, optical microscope, 100× and 400× magnification.

Nasal cytology revealed inflammatory rhinitis with neutrophilic cellularity and few non-degranulated eosinophilic elements (Figure 1).

**Figure 2:**
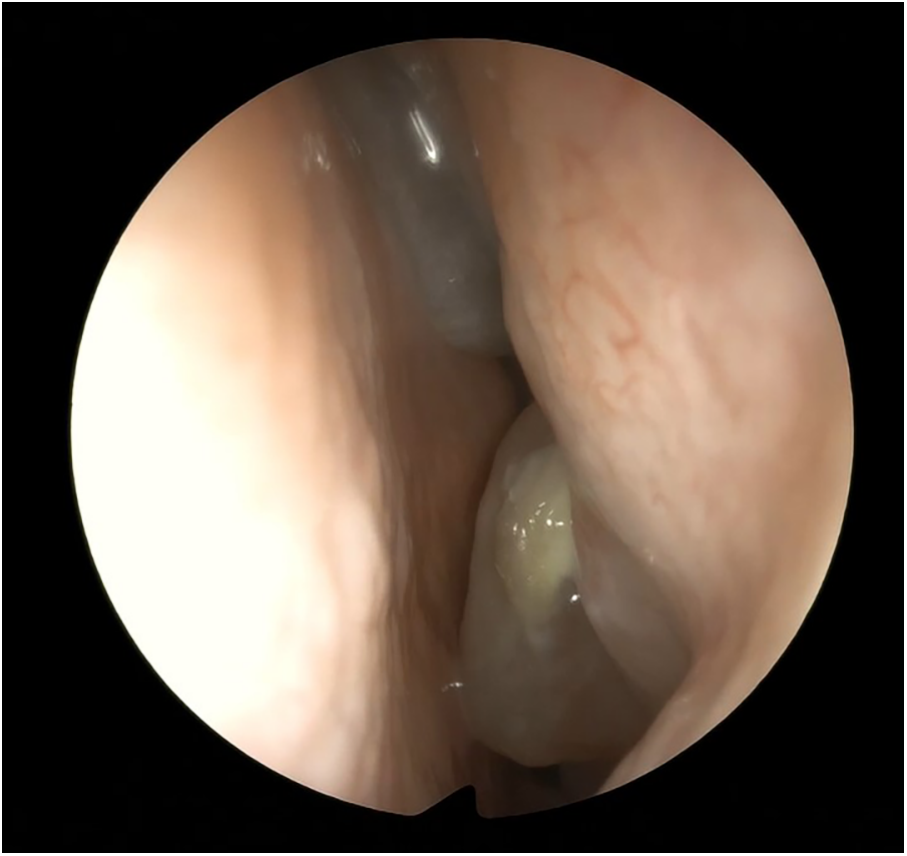
Endoscopic view of the left nasal fossa: polyps from the meatal area and from the anterior ethmoid.

**Figure 3:**
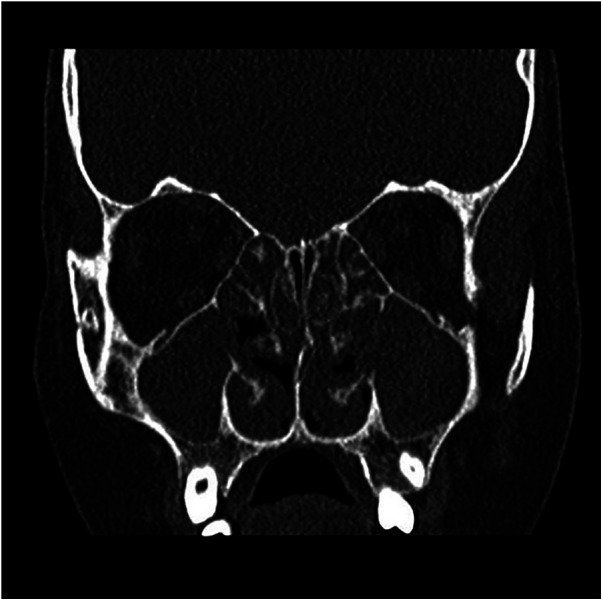
Coronal CT scan: almost complete opacification of the nasal cavities, the ethmoid labyrinth, and the maxillary sinuses.

Nasal fibroendoscopy documented hypertrophy of the lower and middle turbinates with polypoid formations originating from the middle meatus and abundant mucous secretions (Figure 2), with endoscopic Lund–Kennedy scores (8) of 5 and 4 for the right and left nasal cavities, respectively. A paranasal sinus CT scan showed pansinusopathy with complete obliteration of the maxillary sinuses, sphenoid sinus and ethmoid cells, and hypertrophy of the nasal turbinates (Figure 3), with a Lund–McKay score (9) of 10 for both nostrils.

An endoscopic procedure was performed, proceeding with an ethmoidectomy with bilateral sphenotomy and meatotomy, with revision of both maxillary sinuses. A histological examination of the excised material revealed polypoid formations with a ciliated columnar coverage associated with chorion edema containing rare small foci of mononuclear inflammatory infiltrate with rare figures of intraepithelial exocytosis. There was no evidence of eosinophilic granulocytes.

The cytomorphological examination of the material, obtained by “brushing” the nasal mucosa, highlights a hypocellular sample with widespread aspects of squamous metaplasia in which very rare ciliated cells are appreciated with predominantly immobile cilia.

An ultrastructural investigation (TEM) performed on nasal brushing evaluating the ciliated epithelial elements showed the presence of a normal structure of the ciliary axonemes. There were no evident Class 1 modifications of the few assessable axonemes.

Whole exome sequencing (WES) (Twist Bioscience) was performed on proband and his parents and sequenced with paired-end read (150bp) on an Illumina NovaSeq6000 platform. The target regions include the coding exons +/− 25 flanking bases (based on the RefSeq database). We obtained a targeted NGS assay that had a mean coverage of 150× for >97% bases, a specificity of 100%, and a sensitivity of 100%, with a quality score of ≥30. Sequencing data were filtered by a custom *in silico* panel of genes associated with PCD and a nonsense homozygous mutation was identified in a GAS8 gene: c.189G>A p.(Trp63Ter). Sanger sequencing was performed to confirm the homozygous status of the proband and that her parents were heterozygous carriers of the same mutation (Supplementary Figure S1). The list of genes is available in Supplementary Table S1 and includes all genes associated with PCD based on the literature and the Online Catalog of Human Genes and Genetic Disorders (OMIM).

The variant is not present in the general population allele frequency database (gnomAD), nor is it described in the scientific literature. This variant can be classified according to the ACMG guidelines as probably pathogenetic (class 4) (10).

**Figure 4:**
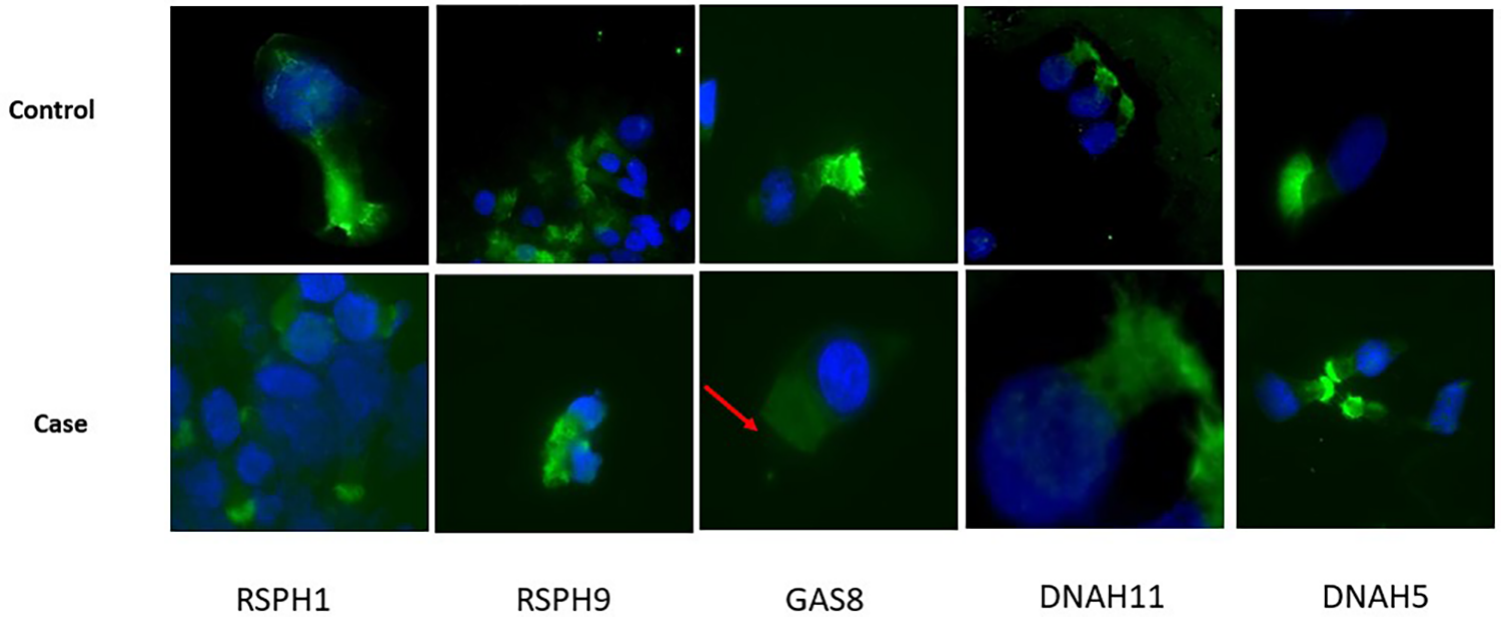
Immunofluorescence analyses. FITC (green) ligand for target protein RSPH1, RSPH9, GAS8, DNAH11, and DNAH5 performed on sample from nasal brushing. The nuclei counterstaining was achieved with DAPI. Acetylated tubulin was not available at the time of tests. Top: the healthy control shows the presence of all the target proteins in ciliated cells; bottom: the patient shows the selective absence of staining for GAS8 in ciliated cells (red arrow).

At the time of diagnosis, the possibility of performing immunofluorescence was not available in our laboratory. However, a protocol was subsequently developed, as previously reported by Shoemark et al. (11), and we were able to confirm the GAS8 deficiency in the current case (Figure 4).

## Discussion

Pathogenetic variants of the GAS8 gene are associated with autosomal recessive primary ciliary dyskinesia (OMIM: 616726). At the time of writing, four mutations have been identified (4, 6).

Olbrich et al. described three GAS8 mutations: (1) homozygous nonsense mutations c.927C>A (p.Cys309Ter) in a 27-year-old Danish man born to non-consanguineous parents; (2) homozygous nonsense mutations c.1069C>T (p.Gln357Ter) in a 6-year-old child from Sri Lanka; and (3) homozygous nonsense mutations c.1000C>T (p.Arg334Ter) in a 21-year-old Libyan woman born to consanguineous parents. All of these patients showed lung disease, chronic rhinosinusitis, and recurrent otitis media but NON-situs inversus. The nasal NO production rate was low and no IDA defects were detectable by TEM and the percentage of misalignment of the ODAs was only slightly increased compared to healthy controls (25% vs. 4.5%–11%) in the two patients examined.

The same nonsense mutation c.1000C>T (p.Arg334Ter) was also observed by Jeanson et al. in a 34-year-old male patient from North Africa along with the nonsense mutation c.547C>T (p.Arg.183Ter) found in a 10-year-old Caribbean boy: these patients, like the Olbrich ones, presented with sinopulmonary syndrome, recurrent otitis media, and NON-situs inversus, with low levels of nasal NO and no IDA defects and slightly increased percentage of axonemal disorganization detected by TEM (25.5%). The patient with nonsense mutations c.1000C>T (p.Arg334Ter) also presented with asthenospermia and Stargardt disease.

In our patient, we identified homozygous nonsense mutation c.189G>A p.(Trp 63Ter) associated with lung disease, recurrent otitis, CRSwNP, NON-situs inversus, low levels of nasal NO, and no IDA defect in assessable axonemes.

Therefore, in summary, biallelic loss-of-function mutations in GAS8 result in a PCD phenotype characterized by a low percentage of 9 + 2 cilia with axonemal disorganization detectable by TEM in approximately 25% of the cilia analyzed, revealing the role of the protein encoded by GAS8 in N-DRC integrity and in the proper alignment of axonemal microtubules in humans (4, 6). At the same time, our new finding expanded the database of PCD pathogenic gene variants, facilitating an accurate diagnosis and genetic counseling for heterogeneous PCD: we identified another recessive loss-of-function GAS8 mutations as a cause of PCD with very subtle abnormalities of ciliary beating and ultrastructure and therefore not easily identifiable by high-speed video microscopy (HSVM) or TEM analysis, just like other PCD variants due to alterations in genes encoding N-DRC proteins (CCDC164, CCDC65, GAS8). Therefore, for the diagnosis of this rare PCD variant, high-resolution immunofluorescence microscopy, which can demonstrate the absence of GAS8 from the ciliary axonemes as well as genetic analyses, is indispensable. Therefore, in patients presenting with a strong clinical suspicion for PCD, we suggest using an extended genetic panel as a diagnostic test, as already recommended by Shapiro et al. (12).

Furthermore, the forms of PCD linked to the variants of the GAS8 gene identified so far are not associated with situs inversus, but with lung disease, otitis, and chronic rhinosinusitis without mention of nasal polyposis (4, 6). However, our patient presented with chronic rhinosinusitis with nasal polyposis, in association with ear and lung disease: to the best of our knowledge, at the time of writing, this is the description of a new variant site in the GAS8 gene, which may constitute a candidate pathogenic variant linked to a form of PCD associated with CRS with nasal polyps.

In this case, nasal polyposis was the tip of the iceberg that led us to the diagnosis of PCD linked to genes that encode subtle ciliary phenotypic anomalies.

In conclusion, the case described here corroborates our decision to follow children with CRSwNP in a multidisciplinary manner (ENT, pulmonologist, allergist, pathologist, pediatrician, and geneticist) because this pathology often hides etiologies that must be recognized. As the symptoms of PCD are non-specific, many patients (particularly those without laterality defects) remain undiagnosed until adulthood, when they are then referred to a pulmonology or ENT clinic because of a significant decline in lung and/or nose function. Earlier awareness may lead to a diagnosis at an early stage and will aid in establishing appropriate follow-up and the introduction of careful management from early life.

## Data Availability

The original contributions presented in the study are included in the article/**Supplementary Material**, further inquiries can be directed to the corresponding author.
