# Supplementary figures and images for: A novel mutation in GAS8 gene associated with chronic rhinosinusitis with nasal polyposis in a case of primary ciliary dyskinesia: a case report
Source: Front Pediatr. 2024 May 30;12:1345265. doi: 10.3389/fped.2024.1345265 (PMC11169881; doi:10.3389/fped.2024.1345265)

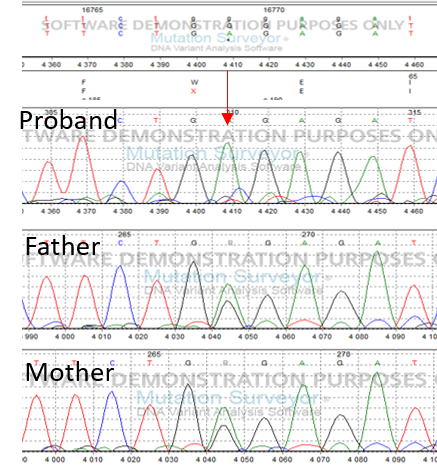

Supplement: Supplementary file 1 [file Image1.png]
