# Supplementary material for: A novel mutation in GAS8 gene associated with chronic rhinosinusitis with nasal polyposis in a case of primary ciliary dyskinesia: a case report
Source: Front Pediatr. 2024 May 30;12:1345265. doi: 10.3389/fped.2024.1345265 (PMC11169881; doi:10.3389/fped.2024.1345265)
Supplement: Supplementary file 2 [file Table1.pdf]

Supplementary Table 1. List of the genes involved in PCD and present in our NGS genes panel

CCDC103	DNAAF1	FLNA	PIH1D3/ DNAAF6
CCDC114/ ODAD1	DNAAF2	FOXJ1	RPGR
CCDC39	DNAAF3	GAS2L2	RSPH1
CCDC40	DNAAF4	GAS8	RSPH3
CCDC65	DNAAF5	HPS4	RSPH4A
CCNO	DNAH1	HYDIN	RSPH9
CEP290	DNAH5	LRRC6	SPAG1
CFAP298	DNAH7	MCIDAS	SPEF2
CFAP300	DNAH8	NEK10	STK36
DNAI1	DNAH9	NME8	TP73
DNAI2	DNAH10	ODAD2	TTC12
DNAJB13	DNAH11	ODAD3	TTC25
DNAL1	DRC1	OFD1	ZMYND10
